# Heavy Metal Rejection Performance and Mechanical Performance of Cellulose-Nanofibril-Reinforced Cellulose Acetate Membranes

**DOI:** 10.1021/acsomega.4c03038

**Published:** 2024-10-02

**Authors:** Seren Acarer-Arat, İnci Pir, Mertol Tüfekci, Sevgi Güneş-Durak, Alp Akman, Neşe Tüfekci

**Affiliations:** † Istanbul University-Cerrahpaşa, Faculty of Engineering, Department of Environmental Engineering, Avcilar, 34320 Istanbul, Turkey; ‡ Istanbul Technical University, Faculty of Mechanical Engineering, Gumussuyu, Istanbul 34437, Turkey; § Centre for Engineering Research, University of Hertfordshire, Hatfield, Hertfordshire AL10 9AB, United Kingdom; ∥ School of Physics, Engineering and Computer Science, University of Hertfordshire, Hatfield, Hertfordshire AL10 9AB, United Kingdom; ⊥ Nevsehir Haci Bektas Veli University, Department of Environmental Engineering, Faculty of Engineering-Architecture, Nevsehir 50300, Turkey

## Abstract

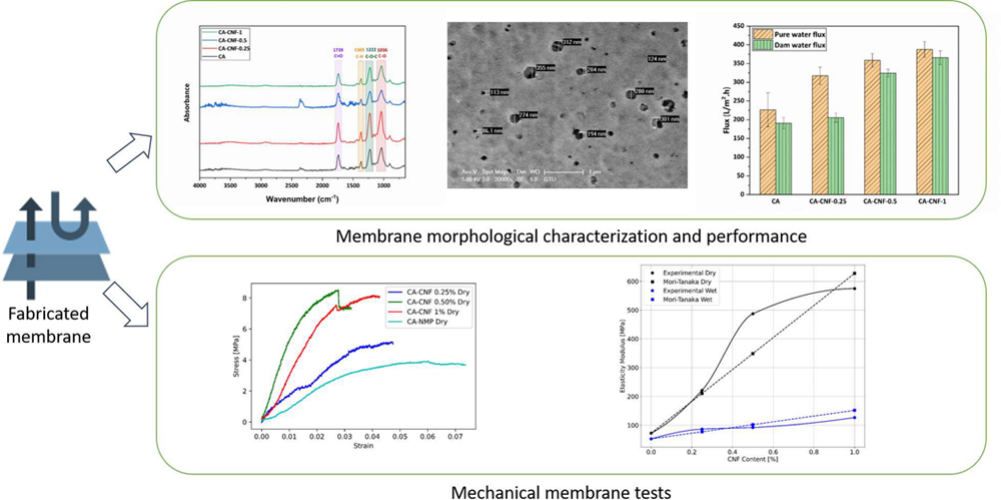

In this research, cellulose acetate (CA) and CA nanocomposite membranes, reinforced with mass fractions of cellulose nanofibrils (CNF), are prepared using the phase separation technique. The membranes are extensively characterized using several techniques: Fourier Transform Infrared (FTIR) spectroscopy confirms the chemical structures, while Scanning Electron Microscopy (SEM) reveals their surface morphology. Mechanical characterization is conducted to explore the mechanical behavior of the membranes under wet and dry conditions through tensile testing. The mechanical properties of CA and CA-CNF membranes are also estimated using the Mori-Tanaka mean-field homogenization method and compared to experimental findings. The flux performance for pure and dam water, assessed at 3 bar, demonstrates that CNF reinforcement notably enhances the CA membrane’s performance, particularly in flux rate and fouling resistance. The CA membrane shows high efficiency in removing Fe^2+^, Ba^2+^, and Al^3+^ from dam water, while CA-CNF membranes exhibit a varied range of removal efficiencies for the same ions, with the 0.5 wt % CNF variant showing superior resistance to surface fouling. Additionally, while CNF increases tensile strength and stiffness, it leads to earlier failure under smaller deformations, especially at higher concentrations. This research provides a detailed assessment of CA and CA-CNF membranes, examining their chemical, structural, and mechanical properties alongside their effectiveness in water treatment applications.

## 1 Introduction

The superior features of membrane processes include low energy requirements, high energy efficiency, low operating cost, no need to use chemicals except for cleaning, simplicity in installation and operation, fast process and superior separation performance. Due to these properties, it is an advanced treatment process used in water and wastewater treatment.^1−3^

Membranes are divided into two classes, polymeric and inorganic, according to the materials they are manufactured from. Inorganic membranes have higher thermal, chemical and mechanical stabilities and longer service life than polymeric membranes; however, the low cost and ease of production of polymeric membranes enable their use in drinking water, wastewater and leachate treatment in laboratory-scale studies and real scale treatment plants.^4−7^ Polymers such as cellulose acetate (CA), poly(ether sulfone) (PES), polysulfone (PSf), polyvinylidene fluoride (PVDF) and polyacrylonitrile (PAN) are examples of widely used polymers in the fabrication of membranes using the phase separation method.^8−10^

CA membranes have unique advantageous properties, such as their raw material being a renewable resource, easy production, low cost, use in all pressure-driven MF, UF, NF and RO membranes, relatively hydrophilic and high affinity for water, relatively low fouling tendency, and nontoxicity.^11,12^

The mechanical behavior of the membrane plays a crucial role in the design process of membrane systems.^13,14^ While not intrinsically strong, polymeric membranes offer the prospect of significant mechanical improvements through targeted nanoreinforcement.^15,16^ Hence, mechanical performance significantly influences the membranes’ overall performance.^17^ The mechanical performance of membranes depends on various parameters. For instance, it is known that adding nanofillers into polymeric membranes significantly changes the mechanical behavior of polymeric membranes.^18,19^ Also, the operating conditions, such as hygrothermal properties of the environment, are shown to be decisive in the mechanical behavior of polymeric materials.^20^ Cellulose, a hydrophilic material, is considerably sensitive to the presence of water, and its mechanics can change significantly.^21,22^

Yang et al. demonstrated that incorporating lignocellulose nanofibrils into cellulose acetate membranes significantly enhances their mechanical strength, hydrophilicity, and antibacterial properties, thereby improving wastewater treatment efficiency.^23^ Similarly, Goetz et al. showed that membranes with cellulose nanocrystals exhibit increased hydrophilicity and effective dye rejection, highlighting their potential in water purification applications in the food industry.^24^ Elele et al. investigated the mechanical properties of polymeric microfiltration membranes, revealing that despite varying pore topologies and polymer properties, the mechanical behavior under stress was consistently robust across different materials, essential for ensuring reliability under operational conditions.^25^

The properties of polymeric membranes used in water and wastewater treatments have become a very popular topic in recent years to improve membrane properties and/or enhance membrane performance by incorporating different nanomaterials with superior properties into a polymeric matrix.^26,27^ The low cost, small size, low density, large surface area, high aspect ratio, good hydrophilicity, high mechanical strength and stiffness, environmentally friendly, and sustainable nanomaterial properties make cellulose nanofibril (CNF) a very good candidate for use as a reinforcement material in polymeric membranes.^28−30^

Cindradewi et al. used CNF as reinforcement to increase the tensile properties of CA film in material strength studies.^31^ Battirola et al. investigated the effects of cellulose nanofibers on the morphology, water flux, and filtration performance of cellulose acetate membranes. The increase in CNF content caused a spongy shape in the SEM images. The pure-water flux and porosity increased as the CNF content increased. It was also observed that the total solids, soluble solids and turbidity parameters decreased.^32^ Gopakumar et al. reported that a PVDF membrane coated with CNF has a lower contact angle (i.e., higher surface hydrophilicity) than a pure PVDF membrane.^33^ Zhang et al. found that PSf/modified CNF membranes had higher surface hydrophilicity, pure water flux, improved tensile strength and increased elongation at break values than pure PSf membranes.^34^

The reinforcement of CA membranes with CNF significantly improves their mechanical stability and longevity by enhancing tensile strength, modulus, and hydrophilicity, as well as by contributing to the formation of a three-dimensional connected porous structure. These improvements are essential for the long-term performance and durability of the composite membrane.^35−37^

In this study, pure CA and nanocomposite CA-CNF membranes reinforced with mass fractions of CNF (0.25, 0.5, and 1 wt %) are manufactured using the phase separation method. The chemical structures are confirmed through Fourier Transform Infrared (FTIR) spectroscopy, surface morphologies are revealed by Scanning Electron Microscopy (SEM), and water contents and mechanical properties of the membranes are investigated. The mechanical performance of the membranes is evaluated through tensile testing under quasi-static loading as well as wet and dry conditions, and the mechanical behavior of the membranes is also modeled using a composite material modeling technique, the Mori-Tanaka Homogenization method. The pure water and dam water fluxes of membranes at 3 bar and the rejection performances of various metals by membranes, including Fe^2+^, Ba^2+^ and Al^3+^ from dam water, are determined by a dead-end filtration system. To the best of the authors’ knowledge, the Fe^2+^, Ba^2+^ and Al^3+^ removal efficiency of CA membranes reinforced with different amounts of CNF from real surface water has not been reported before. In addition, the effect of CNF reinforcement on the mechanical behaviors of CA membranes under both wet and dry conditions are examined for the first time in this study. This study provides a comprehensive evaluation of CA and CA-CNF membranes for water treatment, considering chemical, morphological and mechanical properties as well as their water treatment performance.

## 2 Materials and Methods

### 2.1 Materials

CA (form: powder, average Mn: ∼ 50,000, the extent of labeling: 39.8 wt % acetyl and impurities: ≤ 3.0% water) is purchased from Sigma-Aldrich. NMP (99.5% purity) is purchased from Merck. CNF (form: powder, moisture: ∼ 4 wt %, length: 2–3 μm, width: 10–20 nm) is purchased from Nanografi. Purchased CA and CNF are used directly in membrane production. In accordance with company privacy policies, information regarding the preparation method of CNF from Nanografi could not be provided.

### 2.2 Manufacturing Pure CA and Nanocomposite CA-CNF Membranes

Phase separation is a widely used method for producing commercial polymeric membranes. In this study, pure CA and CA-CNF nanocomposite membranes are prepared using the nonsolvent-induced phase separation (NIPS) method. Table 1 lists the compositions of the casting solutions of the CA-based membranes. To prepare the casting solution of the pure CA membrane, 84 wt % NMP is added to 250 mL flasks, followed by 16 wt % CA. The CA-NMP mixture is mechanically stirred by a magnetic mixer at a 40 °C magnetic stirrer (Wisd, MSH20A) for 48 h until a homogeneous solution is obtained. During the stirring process, the bottles are capped to prevent the solvent from evaporating and impurities from the outside from entering the bottle. For the preparation of casting solutions of CNF-reinforced CA membranes, the required amount of NMP is added to the glass flasks, followed by the addition of the required amount of CNF, which iss then dispersed in NMP with rapid stirring at 40 °C for 10 min. CA (16 wt %) is added to the NMP/CNF mixture and stirred at 40 °C for 48 h. To remove air bubbles from the homogeneous membrane casting solutions, the solution bottles are placed in the degassing mode of an ultrasonic water bath (Weightlab Instruments) at 25 °C for 30 min. After pouring the solutions onto a flat glass sheet, the membranes are spread evenly on the glass sheet using a 200 μm thick casting knife (TQC Sheen, VF2170–261). After approximately 10 s, the glass sheet is immersed in a water bath containing ultrapure water (nonsolvent), and the glass sheet is kept in a water bath for 2 min. The front and back surfaces of the membranes formed as a result of solvent (NMP) and nonsolvent (ultrapure water) exchange are washed three times with ultrapure water, and impurities on the surfaces are removed. The membranes are stored in clean plastic containers with lids containing ultrapure water.

**Table 1:** Composition of the Membrane Casting Solutions

Membrane	CA (wt %)	NMP (wt %)	CNF (wt %)
CA	16	84	-
CA-CNF-0.25	16	83.75	0.25
CA-CNF-0.5	16	83.5	0.5
CA-CNF-1	16	83	1

### 2.3 Characterization of Membranes

#### 2.3.1 FTIR Analysis

The membrane samples are analyzed using FTIR spectroscopy to verify the surface chemistry of the manufactured CA and CA-CNF membranes. The membranes are dried at room temperature for 1 day, and then the FTIR spectra of the membranes are recorded in the range of 4000–650 cm^–1^ using an FTIR spectrometer.

#### 2.3.2 SEM Analysis

The surfaces of the clean and dammed membranes are characterized using an SEM device (Philips XL 30S FEG). Before obtaining surface images of the clean membrane samples stored in ultrapure water, the surfaces of the membrane samples are thoroughly washed with ultrapure water and kept at room temperature until they get completely dry to avoid any impurities in the samples. After draining the dam water, the fouled membranes are placed in Petri dishes with lids and kept until completely dry. The dry membrane samples are made conductive by coating with gold at 10 mA for 120 s using a coater (Quorum SC7620) before the SEM analysis. Finally, the surface images of clean and dam water-filtered membranes are analyzed by SEM at 20000x and 10000× magnification, respectively. Data on the porosity, average pore size (average pore radius) and pore size distribution of the membranes are obtained from SEM images using MATLAB script. All pores on each membrane SEM image are counted in MATLAB.

#### 2.3.3 Water Content

To determine the water content of manufactured membranes, three samples of 3 × 3 cm^2^ are cut from each membrane and stored in ultrapure water. The membrane samples are placed in aluminum weighing dishes and kept in an oven at 60 °C (Nuve EN 500) for 48 h, and their dry weights are determined using a precision balance (Precisa, XB 220A). The membrane samples are then immersed in ultrapure water for 20 s; excess water is quickly removed from the surface of the wet membranes using blotting paper, and the weights of the wet membranes are determined. The water content of each membrane sample is calculated using eq 1. The water content results of the membranes are expressed as the average of three experimental replicates.
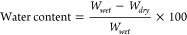
1

Where *W*_*wet*_ and *W*_*dry*_ represent the wet and dry weights (g) of the membrane samples, respectively.

#### 2.3.4 Zeta Potential

The surface charge of the membranes is analyzed using an electrokinetic analyzer (Anton Paar, SurPass). The variation of the surface charge of the membranes with pH is analyzed in the range of pH 3–10 using 1 mM KCl solution.

#### 2.3.5 Contact Angle

A contact angle meter (KSV, CAM 101) is used to determine the surface hydrophilicity of the membranes. Distilled water filled in a syringe is dripped near the center surface of the membranes. Immediately afterward, the angle between the distilled water and the membrane surface is measured. Measurements are performed at room temperature. Three measurements are performed for each membrane, and the results are given as average.

#### 2.3.6 Mechanics of the Membranes

##### 2.3.6.1 Tensile Test

The tensile test is a standardized principal method to assess the mechanical characteristics of materials. The relationship between force and displacement of material can be measured through this experimental approach. Using the acquired data, the stress–strain relation can be plotted, and the elastic modulus, tensile strength, and elongation at the break of the material can be determined. These physical quantities are crucial to understanding the mechanical response of the material and enabling a comparative analysis with other materials quantitatively. Within the context of this research, the strain rate for quasi-static assessments is set at a rate of 1% strain per minute. Each membrane material configuration is tested in both wet and dry (air-dried for 24 h at ambient conditions) to characterize the influence of the water presence for cellulose as a polymeric matrix and reinforcing phase. Aluminum plates are affixed to the specimen’s ends to prevent the slip between the clamps and the sample during testing. The tensile tests are repeated until three consistent data sets are recorded. The tensile tests for the membranes are carried out using the Shimadzu AG-IS 50kN universal testing machine.

##### 2.3.6.2 Material Modeling

Developing new particle-reinforced materials through experiments can be slow and expensive because it involves making samples, running tests, and analyzing the data. Numerical modeling studies offer a faster and less expensive alternative to experimental methods. However, these models typically assume that material distribution is uniform (homogeneous) and material behavior is the same in all directions (isotropic) in order to simplify the calculations. One of the techniques for modeling composites is the Mori-Tanaka mean-field homogenization method. This method is a relatively simple and quick way to predict the elasticity modulus of a composite material based on the properties of matrix and reinforcement materials.

### 2.4 Water Flux and Rejection Performance of Membranes

#### 2.4.1 Water Flux Test of Membranes

Ultrapure water and dam water are passed through the membranes using a dead-end filtration system (Tin Engineering, Turkey) with nitrogen gas to determine the pure water and dam water fluxes of the manufactured water treatment membranes. Samples with diameters of 5 cm are cut from the membranes and placed at the bottom of the filtration setup. After the 300 mL filtration apparatus is completely filled with ultrapure water or dam water, the apparatus is tightly closed using a double open-end spanner. The membrane permeate is then collected in a beaker on a precision balance (AND EJ-610) for 15 min by pressurizing the system to 3 bar using nitrogen gas. The weight displayed on the precision balance is transferred to a computer as a function of time. Using the time-weight data obtained, the pure water and dam water fluxes of the membranes are calculated using eq 2. Pure water flux and dam water flux results of the membranes are expressed as the average of two experimental repetitions.
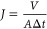
2

Where J, V, A, and Δt represent the membrane flux (L/m^2^.h), filtrate volume (L), membrane area (m^2^) and time (h), respectively.

#### 2.4.2 Rejection Performance Test of Membranes

Dam water is collected from the Akçay Dam in Sakarya, Turkey. The collected water is stored in a clean polyethylene terephthalate (PET) bottle and kept cold before analysis. The 300 mL dam water is filtered from the manufactured membranes using a dead-end filtration setup at 3 bar pressure using the same procedure described in Section 2.4.1, and the permeates are collected in clean beakers. After filtration, Fe^2+^, Ba^2+^ and Al^3+^ in the permeate are analyzed. The physicochemical properties of dam water are listed in Table 2. Measurements to determine the physicochemical properties of dam water are repeated twice.

**Table 2:** Characteristics of Dam Water

Parameter	Unit	Value
pH	-	7.39 ± 0.05
Conductivity	μS/cm	230 ± 2.9
Turbidity	NTU	1.53 ± 0.04
Total Hardness	mg/L CaCO_3_ (°F)	9.70 ± 0.10
Total Organic Carbon	mg/L	1.85 ± 0.12
Fe^2+^	mg/L	0.46 ± 0.04
Ba^2+^	mg/L	0.015 ± 0.002
Al^3+^	mg/L	0.032 ± 0.004

To analyze Fe^2+^, Ba^2+^ and Al^3+^, the analysis sample is first burned with acid to allow the metals to pass into the water in dissolved form, then placed in tubes and left to be read in the Inductively Coupled Plasma (ICP) device.

The rejection performance of the membranes for Fe^2+^, Ba^2+^, and Al^3+^ are calculated using eq 3. The Fe^2+^, Ba^2+^, and Al^3+^ removal efficiencies of the membranes from the dam water are analyzed twice.
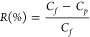
3

In eq 3, *C*_*f*_ and *C*_*p*_ correspond to the contaminant concentrations in the feed and permeate, respectively. R represents the removal efficiency.

## 3 Results and Discussion

### 3.1 Results of FTIR Analysis

**Figure 1:**
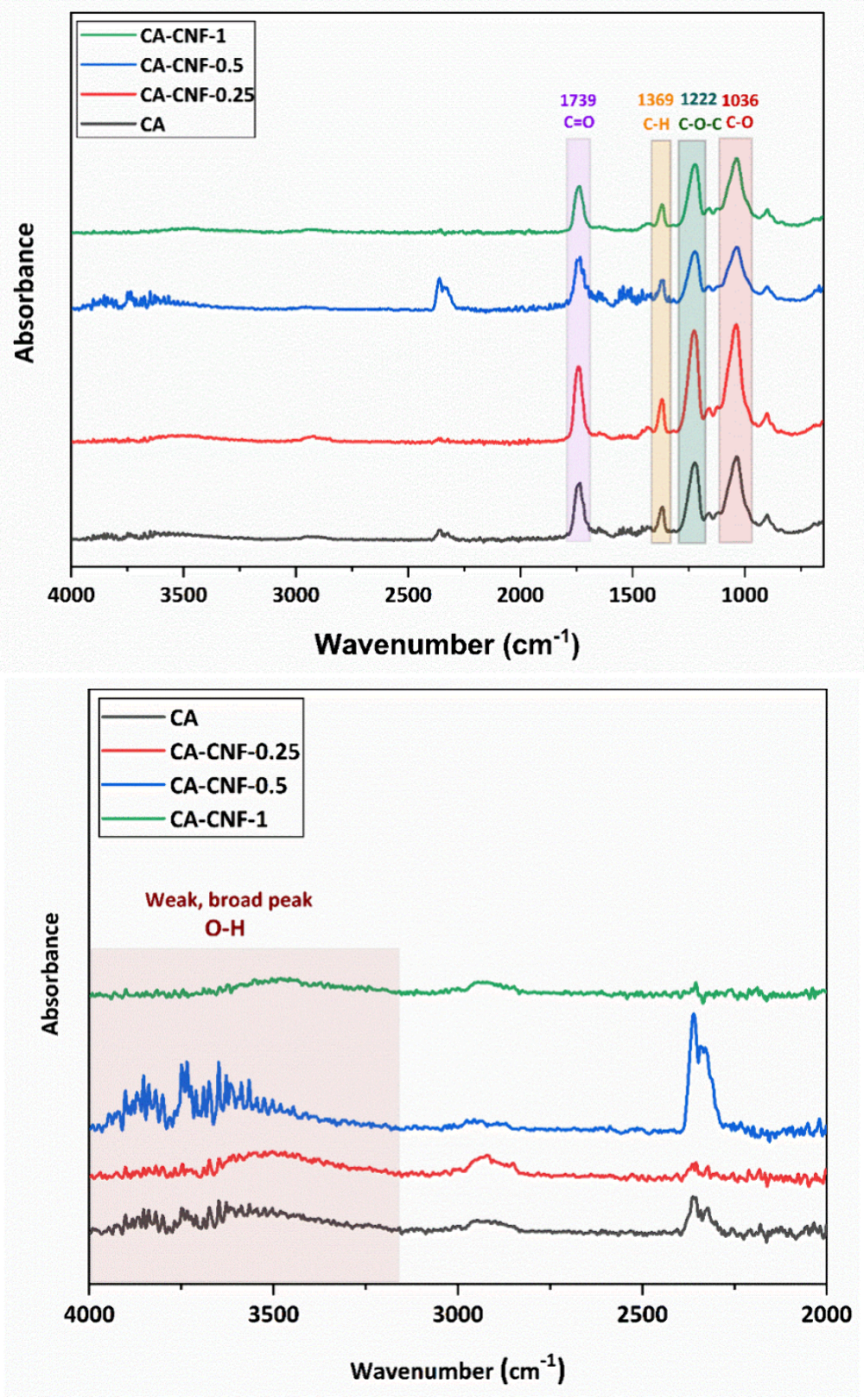
FTIR spectra of membranes: (a) 4000–650 cm^–1^ wavenumber range, (b) 4000–2000 cm^–1^ wavenumber range.

The FTIR spectra obtained from FTIR analysis are performed to verify the chemical structure of the manufactured CA-based membranes, shown in Figure 1. The peaks observed in the spectra of pure CA and nanocomposite CA-CNF membranes in the 4000–650 cm^–1^ wavenumber range are quite similar. In the investigated membranes, peaks are observed, confirming the chemical structure of the membrane, especially at wavenumbers lower than 2000 cm^–1^. The peak at 1739 cm^–1^ corresponds to the C = O stretching vibration, and the peak at 1369 cm^–1^ corresponds to aliphatic C–H stretching vibrations. The two peaks at 1222 and 1036 cm^–1^ can be attributed to C–O–C and C–O stretching vibrations, respectively. Because the peak of the O–H bond presents in the chemical structure of CA and CNF could not be seen in the FTIR spectra in the 4000–650 cm^–1^ range, the FTIR spectra are magnified and plotted in the 4000–2000 cm^–1^ range (Figure 1(b)). In the spectra of all membranes in Figure 1(b), the broad peaks observed in the range of 3200 cm^–1^ 4000 cm^–1^ correspond to the stretching vibration of O–H. In addition, the peaks between 2250 and 2500 cm^–1^ are likely to represent the stretching vibrations of carbonyl groups (C = O) from both cellulose acetate and cellulose nanofibers.^38,39^

### 3.2 Surface Morphology, Porosity, and Pore Size Distribution of Membranes

**Figure 2:**
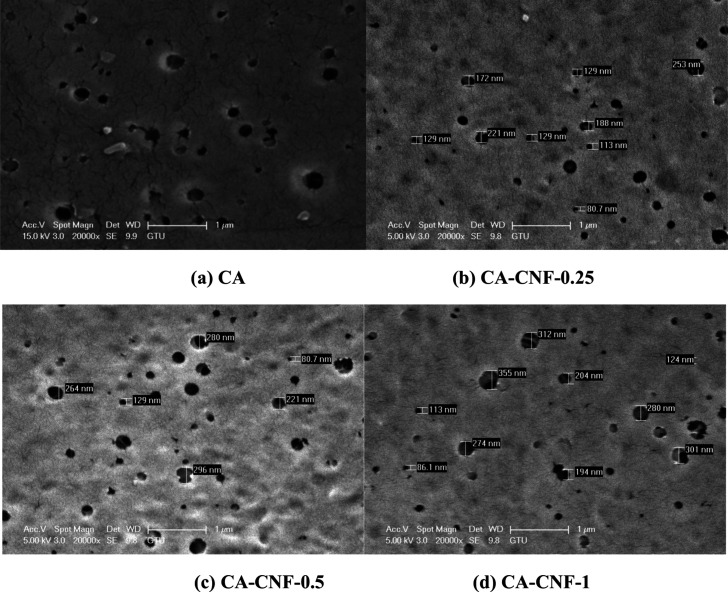
Surface morphology of the membranes visualized via SEM.

To investigate the effect of the CNF reinforcement on the surface morphology of the CA membrane, the surface morphologies of the pure CA and CA-CNF membranes are analyzed by SEM. The manufactured membranes are classified as microfiltration (MF) membranes because the majority of the pores on the surface of both the pure CA and CA-CNF membranes are larger than 100 nm. Although all membranes have a porous surface structure, the surface porosity and size of the pores on the surface of the membranes change with the CNF reinforcement of the CA membrane (Figure 2). The surface properties of the membranes produced by the NIPS method largely depend on the exchange rate between the solvent and nonsolvent during phase separation. The fast exchange between the solvent and nonsolvent leads to higher porosity and larger pore sizes in the membrane, while slow exchange leads to lower porosity and smaller pore sizes. The addition of CNF into the CA-NMP solution destabilizes the solution, and the rate of exchange during phase separation is expected to increase because hydrophilic CNF containing abundant hydroxyl groups has a high affinity for water.^40,41^ However, an increase in solution viscosity causes the exchange between the solvent and nonsolvent to slow down.^41^

**Figure 3:**
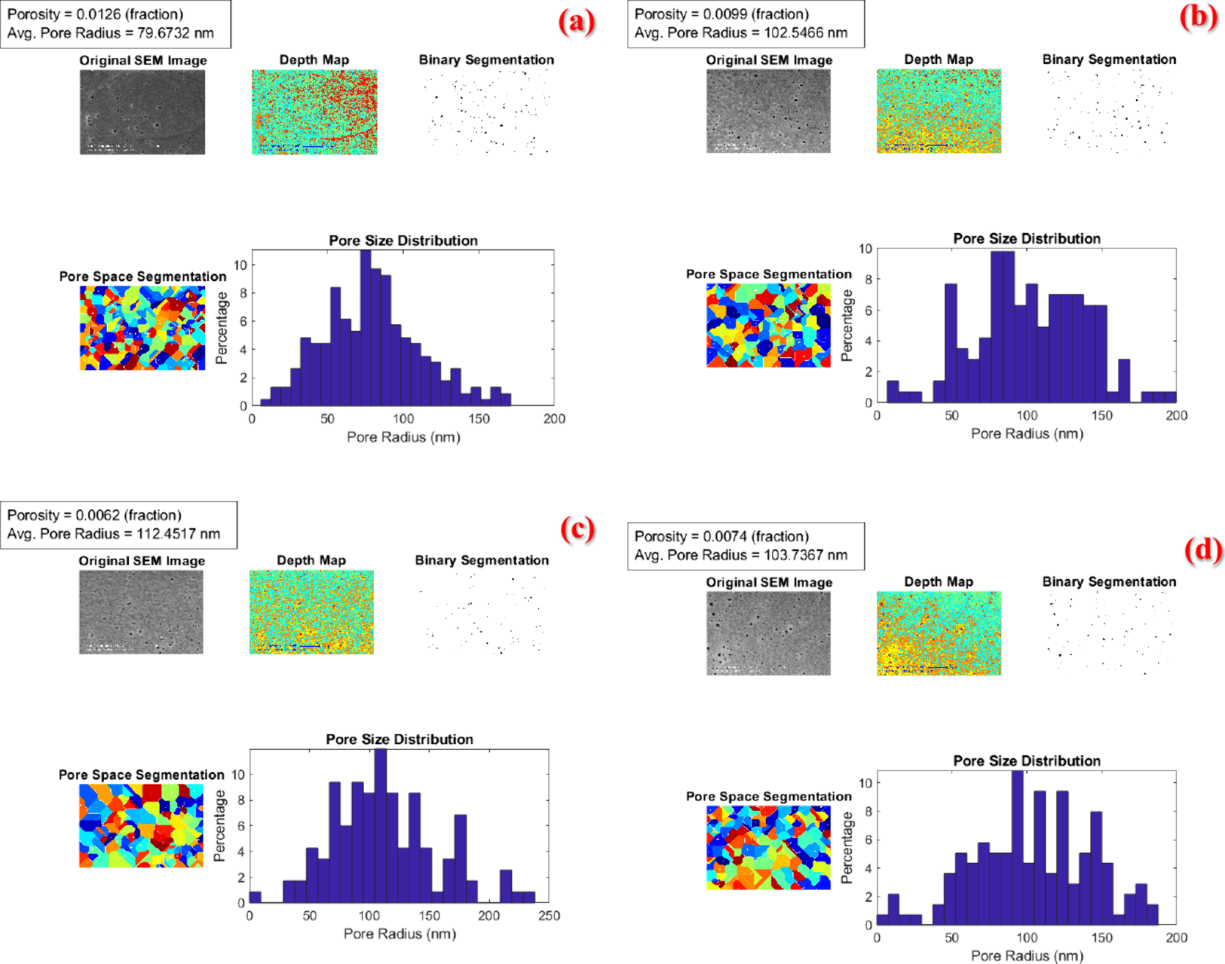
Porosity, pore size distribution, and average pore radius of membranes calculated from SEM surface images in MATLAB: (a) CA, (b) CA-CNF-0.25, (c) CA-CNF-0.5 and (d) CA-CNF-1.

Pore structure, pore size, pore size distribution and porosity can be analyzed from SEM images.^42−45^ This study applies image processing techniques to analyze porosity and pore size distribution in membranes from SEM images using a MATLAB script. The involves the application of image processing algorithms to each image file. Each image is first converted to grayscale to emphasize depth variation, which correlates with porosity. Multilevel thresholding segments the image into distinct depth levels, facilitating the identification of pore spaces. These segmented images are then processed to generate binary maps, highlighting the pores, and subsequently analyzed to determine the porosity and pore size distribution. The results are quantified in terms of average pore radius and porosity percentage, providing a detailed characterization of material porosity. All analysis steps are automated within a MATLAB script, which processes multiple images sequentially and saves corresponding results, including visual plots of the depth maps and pore distributions, in the source image directories. Figure 3 shows the porosity, pore size distribution and average pore size (average pore radius) of the membranes. According to the SEM surface images analyzed in MATLAB, the porosity of CA, CA-CNF-0.25, CA-CNF-0.5 and CA-CNF-1 membranes are 1.26%, 0.99%, 0.62% and 0.74%, respectively. While the surface porosity decreases with the addition of 0.25 and 0.5 wt % CNF to the CA membrane, the surface porosity of the membrane increases with the addition of 1 wt % CNF. The average pore radius of CA, CA-CNF-0.25, CA-CNF-0.5 and CA-CNF-1 are 79.67, 102.54, 112.45, and 103.73 nm, respectively. In this study, there are not very large variations between the average pore diameters of CNF-reinforced nanocomposite membranes. Furthermore, the minimum pore size increased by 42.7%, the maximum pore size increased by 40.3%, and the average pore size increased by 6.7% with increasing CNF reinforcement.^46^ It is clear that the average pore size does not have a lot of variation. The observed increase in the average pore radius of the membrane with 0.25 and 0.5 wt % CNF addition to the CA membrane can be attributed to the hydrophilic CNF enhancing the liquid–liquid exchange rate during phase inversion. On the contrary, the reduction in the average pore radius of the membrane with high CNF addition (1 wt %) can be attributed to the fact that high CNF addition results in an increase in the viscosity of the casting solution. In addition, increasing the amount of CNF in the mixture may lead to decreased pore size due to CNF agglomeration over time.^47^ The average pore radius of the membranes indicates that the membranes produced are MF membranes (Figure 3). According to the pore size distribution of CA membrane (Figure 3a), 50–100 nm sized pores are dominant on the membrane surface. The size of most of the pores on the CA-CNF-0.25 membrane surface is in the range of 75–150 nm. In addition, the distribution percentages of pores in the range of 100–150 nm on the surface of CA-CNF-0.25 membrane are close (Figure 3b). The size of the pores on the surface of CA-CNF-0.5 and CA-CNF-1 membranes are mostly in the range of 50–150 nm (Figure 3c and Figure 3d).

### 3.3 Water Content of Membranes

**Figure 4:**
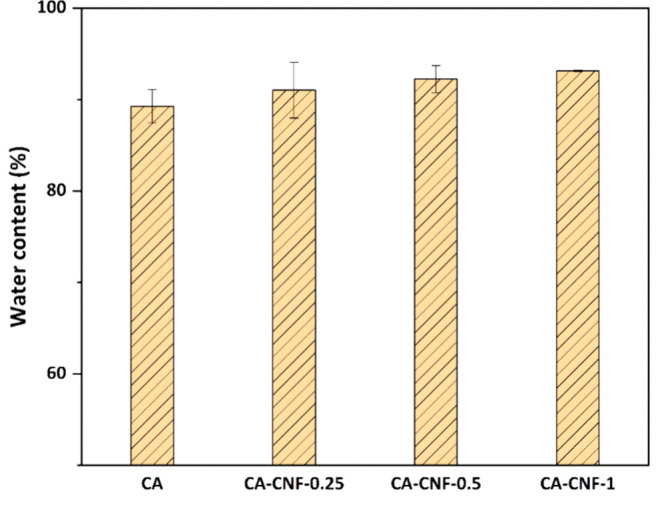
Water content of membranes.

The water content parameter of membranes is important in terms of providing an idea of the water retention capacity of membranes, the amount of water that can pass through the membrane during filtration, and membrane hydrophilicity. In addition, a membrane that cannot retain sufficient water in its structure during filtration indicates inefficient filtration. Figure 4 shows the water content of the CA and CA-CNF membranes. The water content of the CA membrane is 89.26% ± 1.81%, and the water contents of the CA membranes with 0.25%, 0.5% and 1% CNF by weight are 91.01% ± 3.03%, 92.24% ± 1.47% and 93.12% ± 0.09%, respectively. The water content results of the pure CA and CA-CNF membranes are very similar, and the water content of the membranes increases slightly with increasing CNF weight. With the incorporation of hydrophilic CNFs into the CA membrane, as seen in the SEM images, the CNFs increase the size of the pores or porosity on the surface of the membranes (Figure 2), and their high affinity for water enables water to penetrate the membrane structure more efficiently and to be retained more, respectively.

### 3.4 Zeta Potential of Membranes

**Figure 5:**
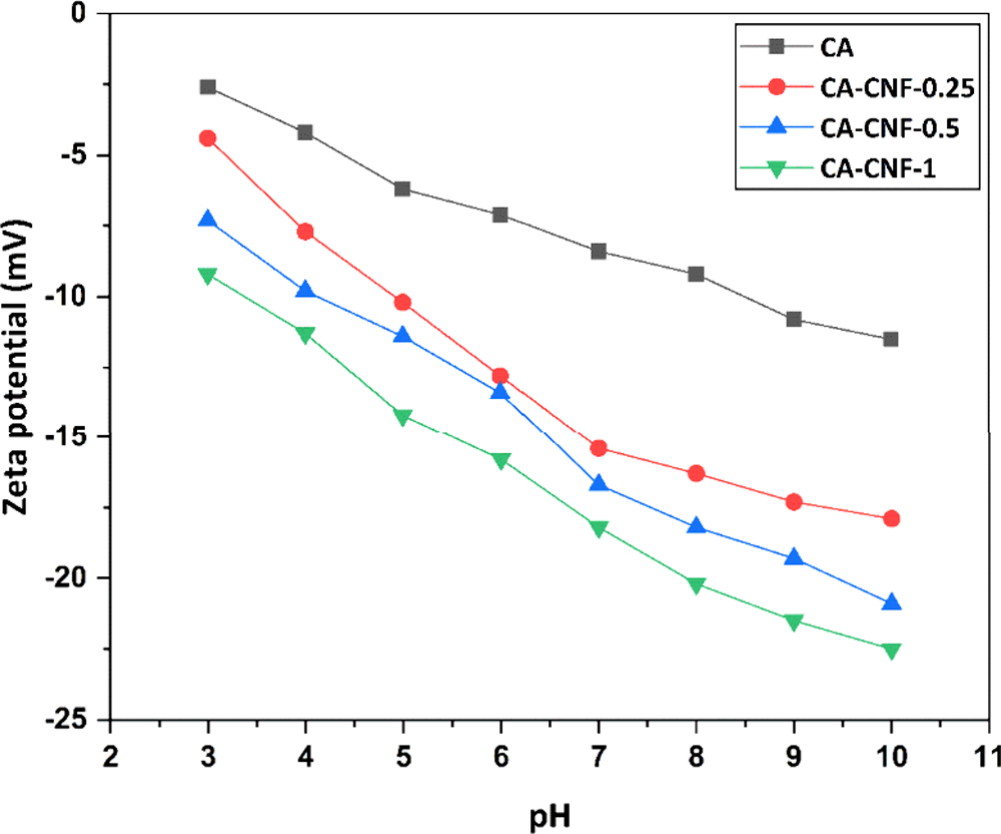
Zeta potential of membrane surfaces as a function of pH.

The surface charge of membranes used in water treatment is of great importance, as it affects the interaction between the membrane surface and pollutants. Figure 5 shows the zeta potential values of the membranes in the range of pH 3–10. The zeta potential of all membranes is negative at all pH values examined. The zeta potential of the membrane exhibits a decreasing trend with increasing incorporation of CNF into the CA membrane. In other words, increasing CNF content causes the surface of the membrane to be more negatively charged. The increase in the carboxylate content on the surface of the CA membrane with CNF incorporation causes the surface to have a more negative charge. Moreover, the surface charge of CNF-reinforced CA-based nanocomposite membranes varies more with pH than the CA membrane. This can be attributed to the pH-dependent protonation–deprotonation behavior of carboxylate groups.

### 3.5 Surface Hydrophilicity of Membranes

**Figure 6:**
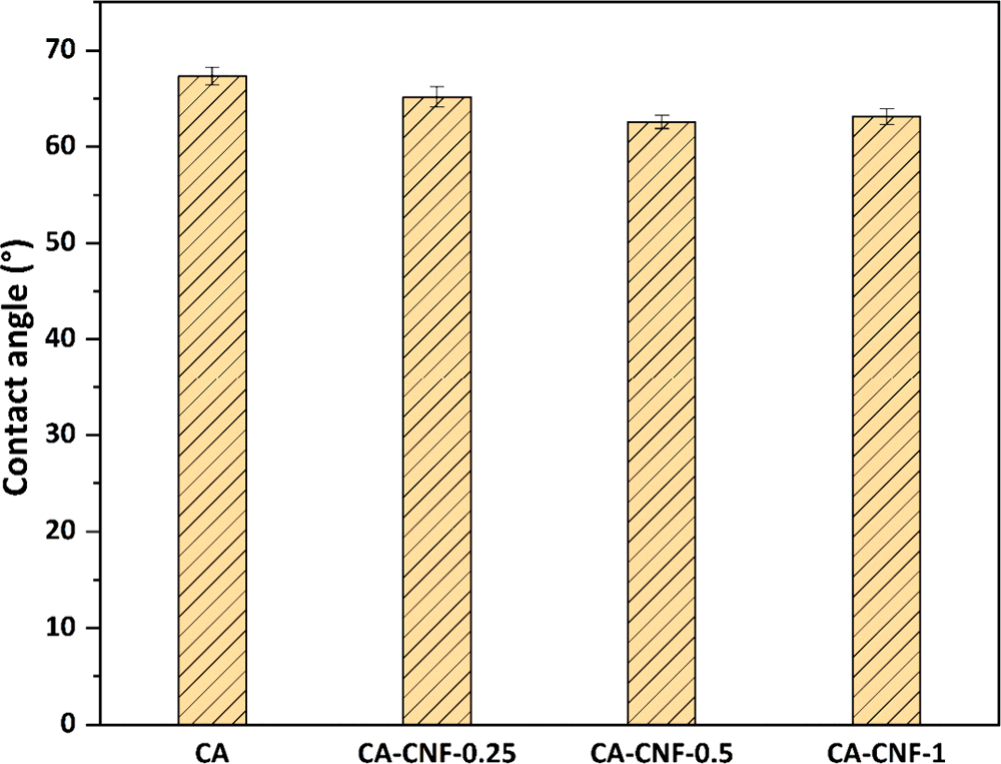
Contact angle values of membranes.

The surface hydrophilicity of membranes is a crucial factor that influences the interaction of the membrane with water and the flux performance of the membrane. The contact angle values of the membranes are presented in Figure 6. The contact angles of the CA, CA-CNF-0.25, CA-CNF-0.5, and CA-CNF-1 membranes are determined to be 67.34 ± 0.9°, 65.18 ± 1.06°, 62.57 ± 0.68°, and 63.15 ± 0.81°, respectively. Given that the contact angles of all membranes are less than 90°, it can be concluded that the membrane surfaces are hydrophilic. The addition of 0.25 and 0.5 wt % CNF to the CA membrane results in a decrease in the contact angle of the membrane and an increase in its hydrophilicity. The abundant hydroxyl groups (−OH) in the structure of CNF enhance the interaction between the membrane surface and water. The increased interaction between the membrane surface and water causes the water to spread more easily on the membrane surface, resulting in a decrease in the contact angle. On the other hand, a slight increase in the contact angle of the high CNF-reinforced nanocomposite membrane is observed. This may be due to the inhomogeneous distribution of CNFs in the polymeric membrane matrix at higher CNF addition and the inability of the hydrophilic −OH groups of all CNFs to come into contact with water due to agglomeration of CNFs. According to the results of this study, 0.5 wt % CNF incorporation into the CA membrane significantly improves the surface hydrophilicity of the membrane by reducing the contact angle of the membrane.

### 3.6 Pure Water Flux and Dam Water Flux of Membranes

**Figure 7:**
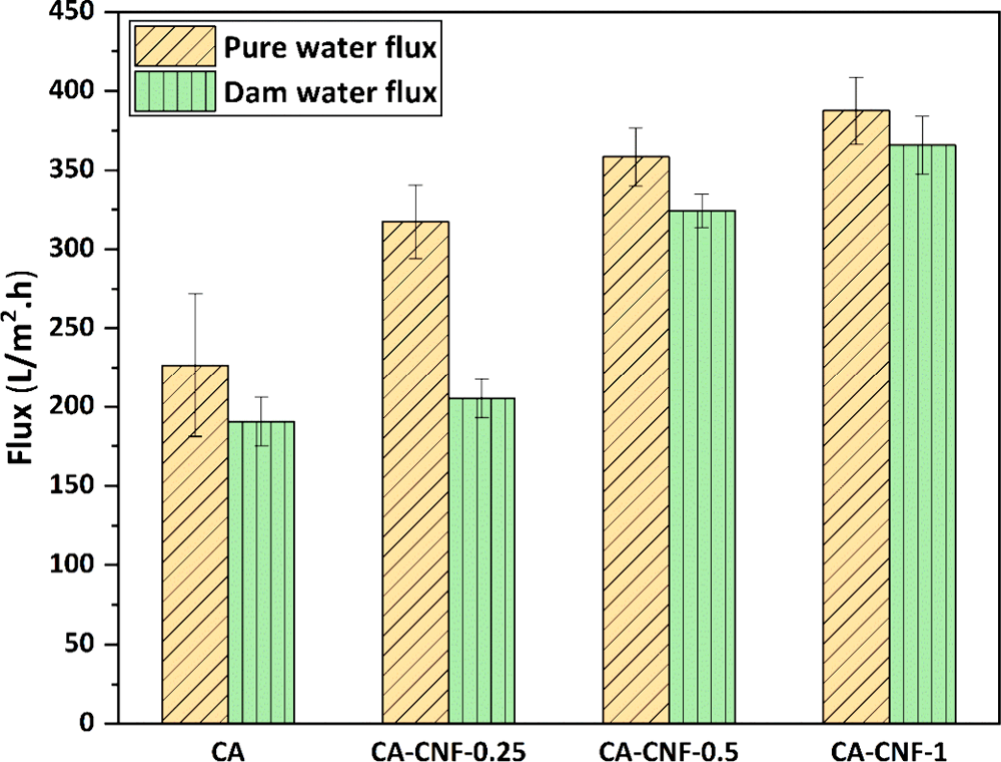
Pure water flux and dam water flux of the membranes at 3 bar.

For membranes used in water and wastewater treatment to provide efficient and high production capacity treatment and low energy consumption, a high pure water flux of membranes is desired. Figure 7 shows the pure water flux and dam water flux values of the CA-CNF nanocomposite membranes. While the pure water flux of the membrane is 226.63 ± 45.43 L/m^2^.h with the addition of CNF to the CA membrane, it increases to 317.23 ± 23.31 L/m^2^.h, 358.41 ± 18.32 L/m^2^.h and 387.48 ± 21.18 L/m^2^.h with the addition of 0.25, 0.5, and 1 wt % CNF, respectively. In other words, 0.25, 0.5, and 1 wt % CNF addition increases the pure water flux of the CA membrane by approximately 40%, 58%, and 71%, respectively. The increase in the pure water flux performance of the membrane from 0.25 wt % to 1 wt % CNF can be explained by the increase in the number of hydroxyl (−OH) groups in the membrane with increasing hydrophilic CNF content. The −OH groups contained in CNFs make them hydrophilic nanomaterials, and CNFs impart hydrophilic properties to the CA membrane when incorporated into the CA-based polymeric membrane. The hydrogen bonding forms between the hydrophilic −OH groups of the CNFs in the structure of the membranes, and the water molecules fed to the membrane contributes to the increase in pure water flux by allowing the water to contact the membrane surface more easily and to flow through the membrane more easily. Therefore, according to the pure water flux results in this study, CNF addition and the presence of up to 1 wt % CNF in the CA membrane allow for faster water treatment and increase the pure water flux performance of the membranes. This is because the inclusion of hydrophilic materials such as CNF increases the hydrophilicity of the membrane surface, allowing the formation of a water layer that facilitates rapid water permeation and increases the flow of pure water.^48,49^ At the same time, membranes with channel structure and uniform pore distribution show high pure water flux.^50^

Surface hydrophilicity, porosity and pore size also affect the water flux performance of membranes. Since the contact angle of CNF-reinforced nanocomposite membranes is lower than the contact angle of pure CA membranes, nanocomposite membranes have higher surface hydrophilicity. High surface hydrophilicity facilitates the penetration of feedwater through the membrane. In addition, as mentioned earlier, although there is no significant difference in the surface porosity of pure CA and nanocomposite membranes, the pores on the membrane surface expanded with increasing CNF content in the matrix of CA membrane. Large pores on the membrane surface contribute to a decrease in the hydraulic resistance of the membrane and faster filtration of water through the membrane. The hydrophilic nature of CNF, the higher surface hydrophilicity of CA-CNF membranes and the larger pores on the surface of CA-CNF membranes resulted in improved flux performance of CA-CNF membranes compared to the flux performance of pure CA membranes.

The lowest dam water flux is 190.62 ± 15.35 L/m^2^.h in the pure CA membrane, whereas the dam water fluxes of the 0.25%, 0.5%, and 1% CNF-added membranes are 205.27 ± 12.10 L/m^2^.h, 324.23 ± 10.55 L/m^2^.h and 365.71 ± 18.25 L/m^2^.h, respectively. Unlike pure water, dam water contains many organic and inorganic substances, so the contaminants in dam water accumulate on the membrane surface and pores, causing clogging of the membrane pores and fouling of the membrane. Because the filtration capacity of a membrane with fouled and clogged pores also decreases, the dam water fluxes of the produced CA and CA-CNF membranes are expected to be lower than that of the pure water flux. Similar to the results of the pure water flux performance of the produced membranes, CA-CNF membranes are better than pure CA membranes in terms of dam water flux performance. With the increasing amount of hydrophilic CNF in the membrane structure, the affinity of the membrane for dam water increases and the passage of dam water through the membrane becomes easier. The increase in flux performance in the filtration of both pure water and dam water, representing real ambient conditions with CNF reinforcement to the CA membrane, provides an increase in flux performance. With CNF-reinforced nanocomposite membranes, higher amounts of clean water can be obtained in a shorter time, and investment and operating costs are reduced as fewer membrane areas are required.

### 3.7 Metal Cation Rejection Performance of Membranes

**Figure 8:**
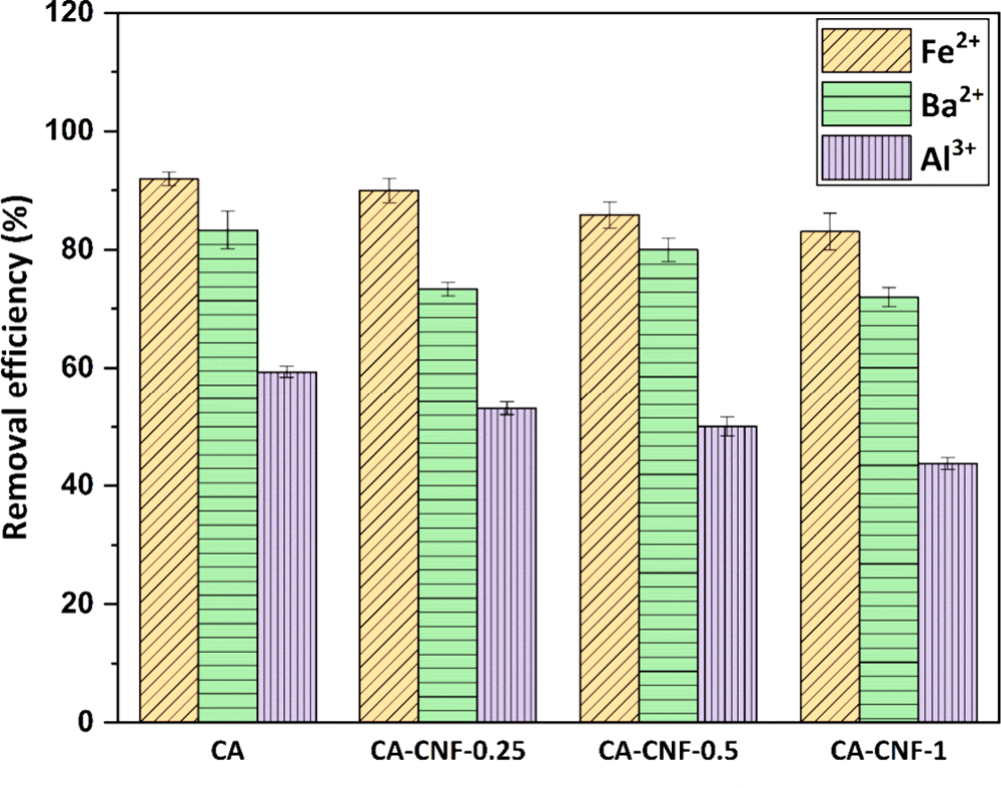
Removal efficiency of metal cations from dam water using membranes.

Figure 8 shows the removal efficiencies of Fe^2+^, Ba^2+^ and Al^3+^ from the dam water through the membranes produced. The concentrations of Fe^2+^, Ba^2+^ and Al^3+^ in the dam water is 0.46 ± 0.04 mg/L, 0.015 ± 0.002 mg/L and 0.032 ± 0.004 mg/L, respectively. The removal efficiencies of Fe^2+^, Ba^2+^ and Al^3+^ from water by the CA membrane are 91.95%, 83.33% and 59.37%, respectively. The rejection performance of all the CA-CNF nanocomposite membranes from water is lower than that of the CA membrane. The Fe^2+^, Ba^2+^ and Al^3+^ varies between 83.04%–90%, 72%–80% and 43.75%–53.12%, respectively.

Membrane properties, properties of metals, membrane-metal interactions and operating conditions affect the metal ion rejection performance of membranes.^51,52^ The low porosity and small pore size of membranes are among the features that increase their rejection performance.^53^ However, the pore sizes of the membranes manufactured in this study are characteristic of the MF membrane, which has the largest pore size among the pressure-driven membranes. Therefore, water-soluble substances that are smaller than the pores of the produced membranes cannot be removed with very high efficiency. The hydrated radii of Fe^2+^, Ba^2+^ and Al^3+^ are 0.428, 0.404, and 0.475 nm, respectively.^54^ Therefore, the size exclusion mechanism of CA and nanocomposite CA-CNF membranes is not the only effective mechanism in removing metal cations from dam water.

The attraction and repulsion interactions between the membrane and metal cations are related to the surface charge of the membrane and the valence of the metal. According to Donnan’s principle, a positively charged membrane surface effectively rejects positively charged metal ions with high valence, whereas a negatively charged membrane surface effectively rejects negatively charged ions with high valence.^55^ In this study, the pH value of the water filtered through the membranes is 7.39 ± 0.05. Studies have shown that CA membranes^56^ and CNF^57,58^ are negatively charged at pH (7.39 ± 0.05), corresponding to the pH of the dam water used in this study. In this study, the zeta potentials of CA, CA-CNF-0.25, CA-CNF-0.5 and CA-CNF-1 membranes at pH 7 are determined as −8.4, −15.4, −16.7 and −18.2, respectively. Since the membrane surface charge is negative at the pH value of the feedwater, positively charged metal ions move toward the membrane. The high-valence Al^3+^ is more attracted by the negatively charged membrane surface than the lower-valence Ba^2+^ and Fe^2+^ and can pass through the membrane pores. Similarly, in a recent study by Liu et al. (2023), the rejection of CuSO_4_, ZnSO_4_, and MnSO_4_ solutions by thin-film nanocomposite membranes with negative surface charges is 91.2%, 94.3% and 92.5%, respectively. In contrast, the removal efficiency for Cr_2_(SO_4_)_3_ is lower at 66.2%. As the Cr^3+^ cation has a higher valence than divalent cations, it is observed that the removal efficiency decreased because of the electrostatic interaction between the membrane surface and the cation.^52^ It is also important to note that as the CNF content in the membrane matrix increases, the surface charge of the membrane becomes more negative (Figure 5). This may cause the metal ions to move more toward the membrane surface due to the electrostatic attraction between the surfaces of the nanocomposite membranes and the metal ions. Therefore, the stronger electrostatic attraction between the metal ions and the nanocomposite membranes may cause a decrease in the removal efficiency of metal ions. It can be concluded that the Donnan effect and the adsorption of metal ions on the membrane surface and/or pores are effective mechanisms for Fe^2+^, Ba^2+^ and Al^3+^ removal from dam water. Furthermore, as observed by the SEM images, the addition of CNF increases the macrovoid’s average diameter. As a result, there is less metal rejection, suggesting that applications needing high flux are better suited for reinforced CNF membranes. This is further supported by its high mechanical and strength.^32^

### 3.8 Surface Morphology of Fouled Membranes

**Figure 9:**
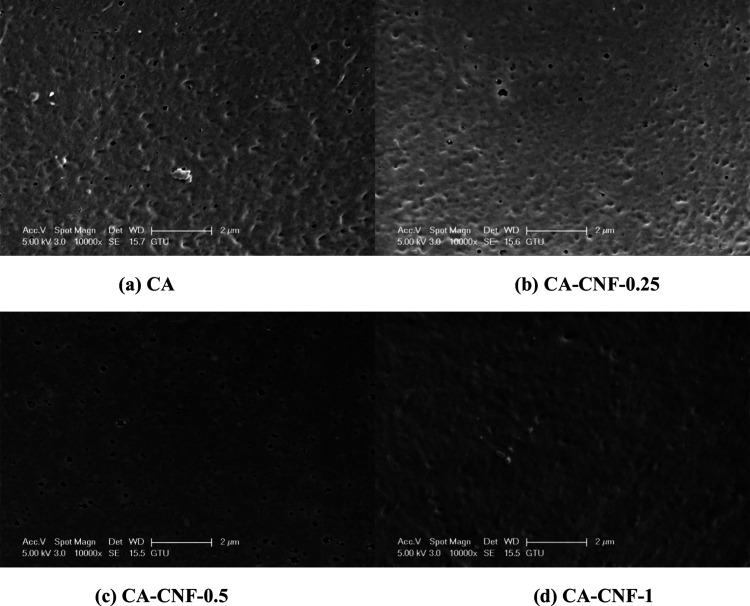
Surface morphology of fouled membranes after dam water filtration.

Figure 9 shows surface images of the fouled membranes where the dam water is filtered. Remarkably, more contaminants accumulate on the surface of the pure CA membrane than on the CA-CNF membranes. This result can be attributed to the relatively more hydrophobic nature of the pure CA membrane surface, which lacks hydrophilic CNF compared to CA-CNF membranes.^33,40^ In CA-CNF nanocomposite membranes, CA membranes with 0.25 and 0.5 wt % CNF reinforcement still have open pores even on the fouled surface, whereas the surface of the CA membrane with 1 wt % CNF reinforcement becomes denser and rougher with fewer open pores. This result is related to the larger pores on the surface of the clean, pure CA and clean CA-CNF-1 membranes compared to the other membranes, as contaminants enter the pores more easily and clog the pores more easily. Based on the fouled SEM surface images, it can be concluded that the CA-CNF-0.5 membrane is the most fouling-resistant membrane, as it exhibits minimal reduction in surface porosity and pore size after dam water filtration.

Contaminants accumulating in the pores and surface of the membranes during filtration cause membrane blockage and fouling. Blockage and fouling make it difficult for water to pass through the membrane and cause a decrease in the flux performance of the membrane.^59^ When the dam water fluxes are measured during the removal of Al^3+^, Ba^2+,^ and Fe^2+^ from the dam water of the membranes, the lowest dam water flux among all membranes belonged to the pure CA membrane. When the SEM surface images of fouled membranes and the dam water fluxes of the membranes are compared, it is determined that the pure CA membrane, which is the membrane most prone to fouling, had the lowest dam water flux (190.62 ± 15.35 L/m^2^.h). The fouling resistance ability and hydrophilic property that CNF imparts to the membrane contributed to the dam water flux of nanocomposite CA-CNF membranes (205.27 ± 12.10–365.71 ± 18.25 L/m^2^.h) being higher than the dam water flux of pure CA membrane during the rejection of contaminants from the dam water by the membrane filtration. If a higher molecular weight material had been chosen as the membrane base material, lower water permeation could be achieved, but higher contaminant removal could be achieved^60,61^ This is due to the increased hydrophilicity of higher molecular weight polymers, which increases the interaction with water molecules and reduces permeability.^62^

### 3.9 Mechanics of the Membranes

#### 3.9.1 Tensile Test

**Figure 10:**
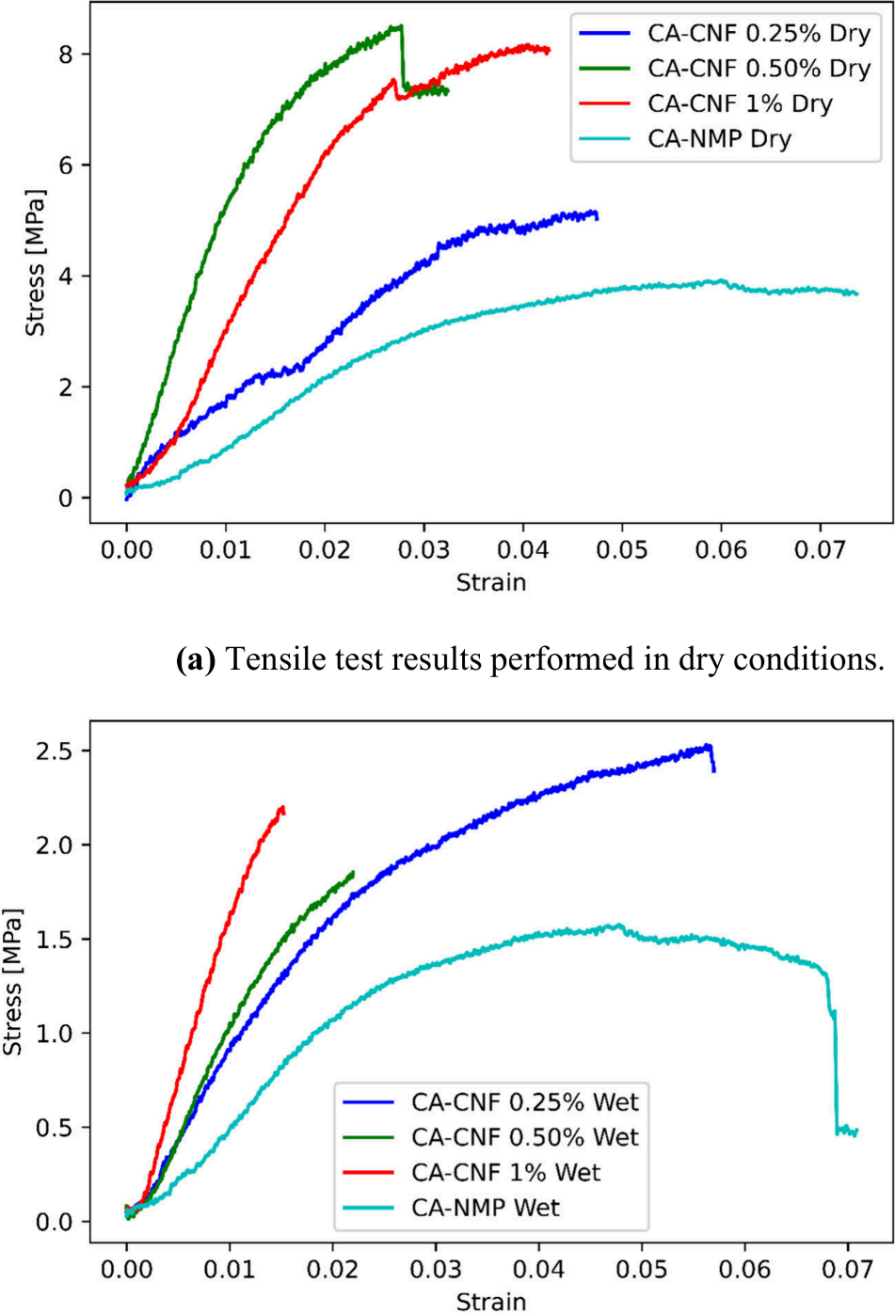
Stress–strain curves of the unreinforced and nanocomposite membranes in dry and wet conditions.

The mechanical properties of CA membranes with different mass fractions of CNF are evaluated under both dry and wet conditions to understand the influence of CNF on the composite’s mechanical performance. In Figure 10, stress–strain curves of the unreinforced and nanocomposite membranes in dry and wet conditions are drawn. From that figure, the change in the mechanical behavior of membranes concerning hygrothermal conditions and nanomaterial concentration can be observed. Figure 10 (a) displays the change in the mechanical behavior of dry membranes, Figure 10 (b) visualizes the wet ones. In order to assess the effect of CNF reinforcement on the mechanical behavior of CA membranes, the measured elasticity modulus, tensile strength and elongation at break values of samples tested under dry conditions are given in Table 3, and samples tested under wet conditions are given in Table 4. The data shown represents the average of three repeated trials, with the associated variability of these measurements also included.

**Table 3:** Mechanical Properties of CA-CNF Composites under Dry Conditions

Specimen Name	Elasticity Modulus (MPa)	Tensile Strength (MPa)	Maximum Strain
CA Dry	72.27 ± 3.52	3.92 ± 0.21	0.0737 ± 0.0046
CA-CNF-0.25 Dry	221.23 ± 9.32	5.17 ± 0.38	0.0474 ± 0.0028
CA-CNF-0.5 Dry	487.15 ± 19.17	8.51 ± 0.57	0.0324 ± 0.0017
CA-CNF-1 Dry	574.86 ± 31.79	8.17 ± 0.72	0.0426 ± 0.0024

**Table 4:** Mechanical Properties of CA-CNF Composites under Wet Conditions

Specimen Name	Elasticity Modulus (MPa)	Tensile Strength (MPa)	Maximum Strain
CA Wet	52.52 ± 3.67	1.58 ± 0.06	0.0709 ± 0.0036
CA-CNF-0.25 Wet	86.51 ± 4.12	2.53 ± 0.11	0.0570 ± 0.0022
CA-CNF-0.5 Wet	92.01 ± 5.88	1.86 ± 0.09	0.0220 ± 0.0009
CA-CNF-1 Wet	126.58 ± 6.36	2.20 ± 0.09	0.152 ± 0.0008

The results acquired from the dry samples suggest a notable rise in the composite’s mechanical properties with CNF addition. The elasticity modulus, the indicator of stiffness, shows a significant rise, even with a minimal 0.25 wt.% CNF addition. It reaches almost eight times the value when pure CA-NMP is compared to a 1 wt.% CNF-reinforced ones.

The tensile strength of the composites, a measure of maximum stress-bearing capacity, exhibits a noticeable increase with CNF inclusion. Remarkably, the highest tensile strength value is captured for the 0.5 wt.% CNF-reinforced membrane, thereby pointing it as an optimum amount for achieving maximum strength increment. However, the tensile strength is observed to change marginally at 1 wt.% CNF concentration, which hints at potential issues such as fiber aggregation or suboptimal fiber-matrix bonding at elevated CNF levels.

On the other hand, elongation at break, a parameter reflecting a material’s ductility, shows a decrease as CNF concentration increases. This is consistent with the general idea that reinforcing rigid nanofibers into a matrix reduces its deformation capability under stress and leads to earlier failure (smaller elongation at break regions) of the composite as the reinforcements tend to cause localized stress concentrations.^63^

When the wet samples are evaluated, the results present an entirely different scenario. Exposure to moisture resulted in an extensive decrease in the mechanical behavior of the membrane samples. Since their hydrophilic nature, CNFs interact with water easily. This interaction causes them to absorb more water, which surrounds the matrix and weakens the fiber-matrix bond. Nevertheless, CA-CNF composites, even in their dampened state, outperformed pure CA-NMP in terms of mechanical performance, highlighting the stable, beneficial effect of CNF.

The elongation at break for CA-NMP and CA-CNF 0.25% remained reasonably constant despite a significant decrease in stiffness and tensile strength in wet conditions. This suggests that the material maintains its ductility even in the presence of moisture. However, higher CNF concentrations, i.e., 0.50% and 1%, indicate a marked transition toward earlier failure (smaller elongation at break regions) when wet.

The reduction in the elasticity modulus and tensile strength of CNF membranes when wet can be primarily attributed to the interactions between water molecules and the CNF structure. When CNF membranes become wet, the water molecules interact with the cellulose, leading to a plasticization effect that lowers the mechanical stiffness and strength of the material. This occurs because water acts as a plasticizer, reducing intermolecular forces and increasing the mobility of the cellulose chains, thereby decreasing the modulus of elasticity and tensile strength.^64^

Moreover, the presence of water can also lead to swelling of the CNF structure. This swelling alters the physical dimensions and internal stresses of the material, further contributing to a decrease in mechanical properties. Swelling due to water uptake generally disrupts the hydrogen bonding network within cellulose, which is crucial for its high strength and stiffness in dry conditions.^65^

#### 3.9.2 Material Modeling

**Figure 11:**
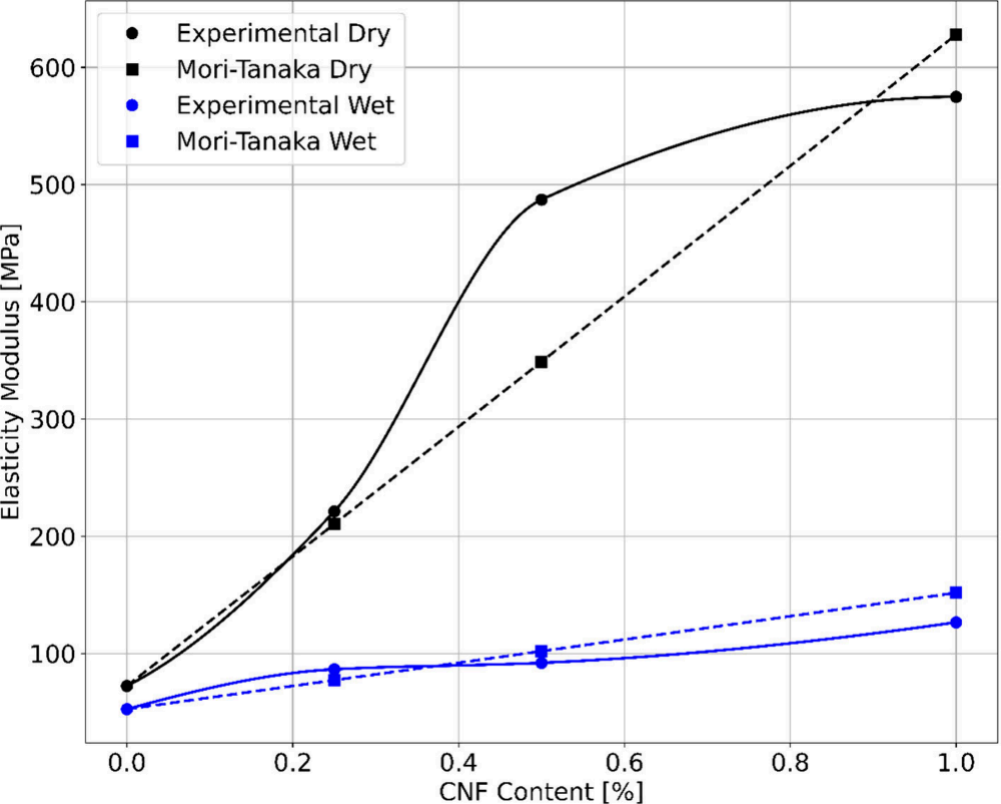
Calculated and measured elasticity modulus values.

The Mori-Tanaka mean-field homogenization method is applied to estimate the mechanical properties of the membranes, followed by a comparative analysis with experimental data. Pure membranes serve as the base matrix phase, with a specific focus on investigating the impact of reinforcing CNF particles on the composite material. Through this method, the elasticity modulus values of the composite membranes are derived. The results are presented as the analytical output in Table 5 and Figure 11.

**Table 5:** Experimental and Theoretical Determination of the Elasticity Modulus of Membranes

Condition	Dry		Wet	
Reinforcement Ratio	Experimental Elasticity Modulus	Mori-Tanaka Elasticity Modulus	Experimental Elasticity Modulus	Mori-Tanaka Elasticity Modulus
0.25%	221.23	210.41	86.51	77.20
0.5%	487.15	348.91	92.01	101.96
1%	574.86	627.88	126.58	151.76

Following the analysis, it is determined that the calculated elasticity modulus values varied under wet and dry conditions for different weight percentages of CNF-reinforced CA membrane structures. Specifically, for membranes reinforced with 0.25% by weight of CNF, the elasticity modulus is 77.207 MPa under wet conditions and 210.41 MPa under dry conditions. Similarly, for membranes reinforced with 0.5% and 1% by weight of CNF, the elasticity modulus increased to 101.96 and 151.76 MPa under wet conditions and to 348.91 and 627.88 MPa under dry conditions, respectively.

The analysis revealed a consistent trend of increased elasticity modulus with higher CNF reinforcement levels in the CA membrane. For instance, adding 0.25% CNF resulted in a 47% and 191.14% increase in elasticity modulus under wet and dry conditions, respectively. Similarly, the incorporation of 0.5% and 1% CNF led to more significant enhancements, with elasticity modulus increases of 94.14% and 188.96% under wet conditions and 382.79% and 768.8% under dry conditions, respectively. From these data, it can be concluded that the 1% by weight CNF-reinforced dry CA membrane exhibited the highest rigidity under mechanical loading among the tested membranes.

## 4 Conclusion

In this study, the mechanical behavior of CA composites with incremental CNF concentrations in both dry and wet conditions are considered. Under dry conditions, the increase in the mass fraction of CNF causes an increase in the elasticity modulus of the CA matrix, even tripling at a mere 0.25% CNF content. This increment is supported by existing literature highlighting CNF’s reinforcing capabilities, driven by its high aspect ratio and inherent rigidity. In terms of tensile strength, the composite with 0.5 wt % CNF exhibits peak mechanical performance, suggesting an ideal CNF concentration for optimal tensile strength. However, with CNF concentrations rising to 1%, the tensile strength begins to show signs of saturation. The material’s elongation at break regions reduces as CNF concentration increases, reflecting the trade-off between stiffness and ductility upon integrating rigid nanofibers.

Contrarily, when the test results under wet conditions are evaluated, the composites’ mechanical behavior changes slightly due to CNF’s hydrophilic tendencies facilitating water uptake, thereby limiting the fiber-matrix bond in the structure. Even under these dampened conditions, CNF-reinforced composites show superior mechanical resilience compared to pure CA-NMP ones, emphasizing CNF’s positive influence. While elasticity modulus and tensile strength registered a considerable decrease upon wetting, elongation at break region for CA-NMP and the 0.25 wt % CNF membrane remained relatively stable. However, at higher CNF concentrations, there is a marked increase in elongation at break regions in the wet state. Mechanical modeling studies also verify the increase in elasticity modulus with the CNF reinforcement increment in the matrix both in wet and dry conditions.

To summarize, CNF emerges as a potent reinforcement for CA and significantly enhances its mechanical behavior. Nonetheless, it is imperative to exercise caution in wet working environments, as CNF’s hydrophilic properties could potentially compromise the composite’s effectiveness.

CA and CNF-reinforced nanocomposite CA membranes (CA-CNF) are fabricated and characterized using the phase separation method. The Fe^2+^, Ba^2+^ and Al^3+^ removal performances of the membranes from flux and dam water at a pressure of 3 bar are investigated. The results of the study show that the water content of the membrane, pure water flux performance and dam water flux performance representing the natural aquatic environment are significantly improved because of the increase in the hydrophilicity of the membrane with the addition of up to 1 wt % CNF in CA membranes. The Fe^2+^, Ba^2+^ and Al^3+^ removal performance of CA membrane is slightly higher than CNF-reinforced nanocomposite CA membranes. Tests with dam water show that the membrane surface becomes more resistant to fouling at reinforcements of up to 0.5 wt % CNF addition to the CA membrane. When the characterization and performance results of the membranes produced in this study are evaluated in general, a high pure water flux (358.41 ± 18.32 L/m^2^.h), high dam water flux (324.23 ± 10.55 L/m^2^. h), good metal ion rejection efficiency (85.86 ± 2.25%, 80 ± 1.95% and 50 ± 1.55% for Fe^2+^, Ba^2+^ and Al^3+^, respectively), and high surface fouling resistance, it is suggested that the CA-CNF-0.5 membrane is the most suitable for use in water treatment.

Within the scope of this research, dam water with low metal concentration is filtered through the produced membranes and the removal efficiency of metals is investigated. In further studies, filtering wastewater with high metal concentration and/or solutions with high metal concentration through CA-CNF membranes may contribute to a better understanding of the metal removal mechanism of CA-CNF membranes.

## Data Availability

No data sets were generated or analyzed during the current study.
